# Quince peel polyphenolic extract blocks human colon adenocarcinoma LS174 cell growth and potentiates 5-fluorouracil efficacy

**DOI:** 10.1186/s12935-016-0276-7

**Published:** 2016-02-02

**Authors:** Ichrak Riahi-Chebbi, Meriam Haoues, Makram Essafi, Ons Zakraoui, Sami Fattouch, Habib Karoui, Khadija Essafi-Benkhadir

**Affiliations:** 1 Laboratoire d’Epidémiologie Moléculaire et de Pathologie Expérimentale Appliquée Aux Maladies Infectieuses (LR11IPT04), Institut Pasteur de Tunis, 13 Place Pasteur, BP 74, 1002 Tunis–Belvédère, Tunisia; 2 Laboratoire de Recherche sur la Transmission, le Contrôle et l’Immunobiologie des Infections (LR11IPT02), Institut Pasteur de Tunis, 1002 Tunis, Tunisia; 3 Institut National des Sciences Appliquées et de Technologie (INSAT), Université de Carthage, Tunis, Tunisia; 4 Université de Tunis El Manar, 1068 Tunis, Tunisia

**Keywords:** Quince peel polyphenolic extract, *Cydonia oblonga* Miller, Colon cancer, Anti-tumoral effect, Mechanism of action, 5-Fluorouracil

## Abstract

**Background:**

Development of alternative cancer-specific drugs would be of paramount importance to overcome toxicity toward normal tissues and tumor resistance. Here, we investigated the potential anti-tumoral effect of peel (Peph) and pulp polyphenolic extracts from the Tunisian quince *Cydonia oblonga* Miller on both no-tumorigenic cells NIH 3T3 Fibroblasts and HEK 293 cells and human colon adenocarcinoma LS174 cells.

**Methods:**

Cell proliferation and cytotoxicity were measured with MTT and LDH assays respectively. Cell cycle distribution and the apoptosis levels were assessed by flow cytometry. Intracellular reactive oxygen species (ROS) levels were determined using the fluorescent probe CM-H2DCFDA. Western blot was used to further characterize cell death and analyze the signaling pathways affected by Peph treatment. The expression level of VEGF-A was evaluated by real time quantitative PCR and further verified by quantifying the secreted cytokines by enzyme-linked immunosorbent assay.

**Results:**

We found that Peph extract displayed the highest anti-proliferative effect specifically on LS174 cells. However, each Peph phenolic compound alone did not exhibit any anti-proliferative activity, suggesting a synergistic effect of phenolic molecules. Such effect was associated with a cell cycle arrest in the G1/S phase, a caspase-independent apoptosis and an increase of the ROS production. Peph extract inhibited the pro-survival signaling pathway NFκB and suppressed the expression of various cellular markers known to be involved in cell cycling (cyclin D1) and angiogenesis (Vascular Endothelial Growth Factor, VEGF). Interestingly, the combination Peph extract and 5-FU exerted synergistic inhibitory effect on cell viability.

**Conclusion:**

These data propose the quince Peph extract as a promising cost effective non toxic drug to employ alone or in combination with conventional anti-colorectal cancer. Moreover, quince rich regimen may prevent the development and the progress of colon cancer.

**Electronic supplementary material:**

The online version of this article (doi:10.1186/s12935-016-0276-7) contains supplementary material, which is available to authorized users.

## Background

Colorectal cancer (CRC) is the second most fatal and the third most diagnosed type of cancer worldwide. Despite having multifactorial causes, most CRC cases are mainly determined by dietary factors [1]. Nutrition has been estimated to cause more than one-third of cancer deaths, and that dietary factors are responsible for 70–90 % of all cases [2].

Plants have proved to be an important source of anti-cancer drugs [3]. Polyphenols are secondary metabolites widely present in plant kingdom that play promising role in cancer prevention and therapy [4]. Several studies using cancer cell lines and animal models of carcinogenesis have shown that a wide range of polyphenols possess anticancer properties including initiation of apoptosis through the regulation of cell death pathways, the suppression of cancer cell proliferation and metastasis through inhibition of anti-apoptotic molecules, and cell cycle arrest [5]. Although polyphenols are generally recognized as antioxidants, they also act as prooxidants inducing growth arrest and cell death through increasing ROS production [6].

The most important signaling pathways regulating cell proliferation and survival implicated in colorectal cancer involve Wnt/β-catenin, phosphatidyl-inositol-3-kinase (PI3 K), growth factor receptors/Ras/mitogen-activated protein kinases (MAPK), JAKs/STAT3 and especially nuclear factor κB (NF-κB) [7]. Induction of NF-κB transcription factor, caused by extracellular stimuli, passes through IκB kinase α (IKKα) and/or IKKβ activation [8]. The phosphorylation of IκB inhibitory proteins by IKK activated complex induces ubiquitination and degradation of the IκBs. The dissociated NF-κB complex relocates to the nucleus where it binds to DNA promoter region and activates genes involved in several cellular activities like cell growth, survival, angiogenesis, migration and metastasis [9]. Two major target genes of NF-κB, cell cycle cyclin D1 and Vascular Endothelial Growth Factor (VEGF), are known to play a vital role in tumor progression [10]. This perfectly correlates with the fact that inhibition of NFκB activity in colorectal cancer cells dramatically reduces cell growth in vitro and in vivo [11].

Considering this, several dietary natural phytochemical compounds have been found to be potent inhibitors of NF-κB pathway with anticarcinogenic properties [12]. *Cydonia oblonga* Miller (quince) is recognized as a good and low-cost natural source of different classes of phenolic compounds responsible for its anti-oxidant, anti-ulcerative and anti-microbial activity [13, 14]. We have previously showed that quince peel polyphenols have a potent anti-inflammatory effect in LPS-stimulated human macrophages and that such effect pass through inhibition of NF-κB activation [15]. Moreover, quince polyphenols were reported to present antiproliferative activity in human cancer cells [16]. Notwithstanding these various studies, the anti-tumor effect with mechanisms of action of *Cydonia oblonga* Miller has never been assessed. Here, we investigated the anti-colon cancer activity of polyphenolic extract from the Tunisian quince (*Cydonia oblonga* Miller). We found that both quince peel polyphenolic extract (Peph) and pulp polyphenolic extract (Puph) inhibits viability of human colon adenocarcinoma LS174 cells. However, Peph present the most potent antitumor effect through the blocking of cell growth and the induction of apoptosis and a cell cycle arrest accompanied with an increase of reactive oxygen species (ROS) production. Moreover, Peph extract significantly enhances the anti-cancer effect of 5-fluorouracil. This study suggests that Cy*donia oblonga* Miller phenolic extract may have therapeutic applications for colon cancer treatment and that a quince rich regimen may prevent the development and the progress of colon cancer.

## Results

### Quince polyphenols inhibit human colon cancer LS174 cells viability

**Fig. 1:**
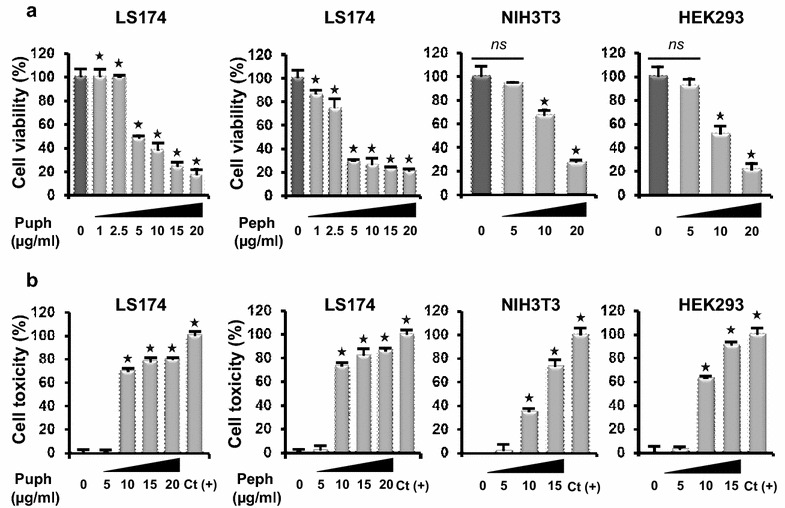
*Cydonia oblonga* Miller polyphenolic extract reduces LS174 cell viability in a dose-dependent manner. **a** Non-tumourigenic cells HEK293, NIH 3T3 and Human colon adenocarcinoma LS174 cells were treated for 72 h with increasing concentrations (1–20 µg/ml) of *Cydonia oblonga* Miller peel (Peph) and pulp (Puph) polyphenolic extract. Cell viability was determined by MTT assay. **b** LDH release from HEK293, NIH 3T3 and LS174 cells was determined by using the LDH assay after 72 h of treatment with different concentrations (5–20 µg/ml) of Peph and Puph. Results were normalized to each control in percentage and represented as mean ± SE of three independent experiments, each performed at least in triplicate. Statistical differences were analyzed with Student’s t test (*p < 0.05 as compared to the control, *ns:* non significant)

**Fig. 2:**
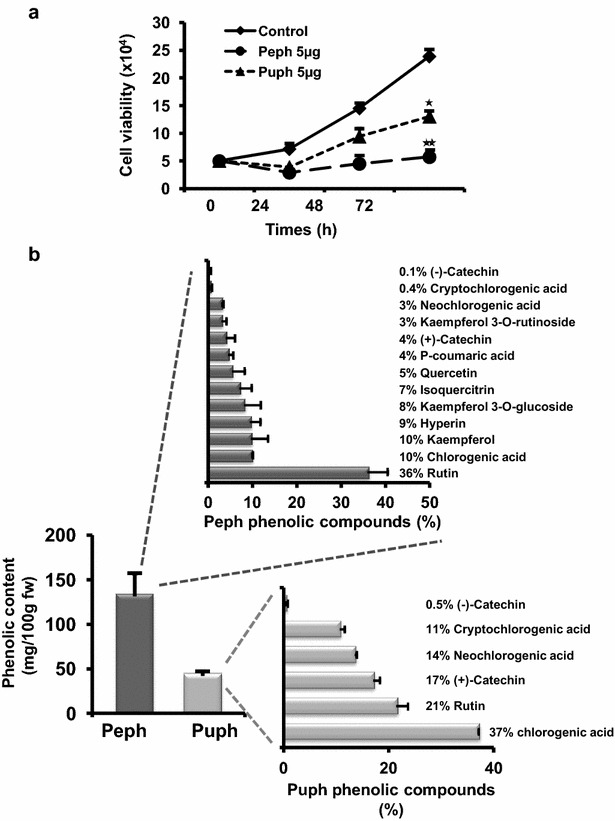
The phenolic profile of Peph and Puph extracts and their effects on LS174 cell viability. **a** Cell viability of LS174 cells treated with 5 µg/ml of quince polyphenolic extracts (Peph and Puph) for 72 h was determined by MTT assay. **b** Each phenolic compound in peel and pulp was identified and quantified using high-performance liquid chromatography with diode-array detection (HPLC–DAD) coupled on line to a mass spectrometer (MS). Based on a concentration [mg/100 g of the fresh weight (fw)] and combination of retention time and spectral matching of compounds, the percentage (%) of each phenolic molecule was calculated

To investigate whether the polyphenolic extracts from the peel (Peph) and pulp (Puph) of quince *Cydonia oblonga* Miller [13] exhibit anti-tumor activites, MTT assay was employed to assess their effects on cell viability in LS174 colon adenocarcinoma cells. Interestingly, we found that increasing concentrations (1–20 µg/ml) of Peph and Puph polyphenolic extracts significantly reduced viability of LS174 cells in a dose-dependent manner after 72 h exposure (Fig. 1a). However, at concentrations higher than 5 µg/ml, the antiproliferative effects on LS174 cells were accompanied by a concomitant increase in LDH activity and cell toxicity (over 70 % of LDH leakage compared to positive control (100 % toxicity, Triton 1 %), as shown by LDH assay (Fig. 1b). Thus, for further investigation, we have chosen the concentration of 5 µg/ml of quince polyphenols that did not affect the viability of non-tumorigenic cells, the NIH 3T3 Fibroblasts and HEK 293 cells (Fig. 1a) and that caused more than 50 % inhibition of LS174 cell viability without any measured cell toxicity. We next followed the kinetic, over 3 days, of the inhibitory effect of 5 µg/ml of Peph and Puph phenolic extracts, on LS174 cell viability. As shown in Fig. 2a, both polyphenolic fractions induced a time-dependent cancer cell growth decrease with an inhibitory effect of Peph extract significantly higher (70 %) than the one exerted by Puph extract (48 %). Analysis of the phenolic composition of each extract has also revealed that phenolic content in the Peph is three times higher than the one of the Puph (Fig. 2b).

### Quince peel polyphenolic extract inhibited-growth of LS174 cells involves total phenolic molecules

In order to identify the active phenolic compounds behind the antiproliferative effect of Peph polyphenolic extract, the effects of its thirteen phenolic constituents were evaluated against the human tumourigenic colon cell line, LS174. All compounds where tested at equivalent concentrations to that present in 5 µg/ml of the total peel polyphenolic extract. Surprisingly, at this concentration, each phenolic compound failed alone to induce significant effect on LS174 cell viability (Table 1). Moreover, we found no significant inhibitory effect, on LS174 cells growth, when we tested the 78 possible combinations of two isolated phenolic compounds (Table 2). We have further extended our analysis to assess the effect of 200 combinations of three compounds or more. However, we did not succeed to reconstitute the effect obtained with the whole Peph extract (Additional file 1: Figure S1), suggesting that the Peph-inhibited proliferation of LS174 cells relies on the synergetic effect of the total polyphenolic molecules.

**Table 1:** Effect of Peph phenolic compounds on proliferation of LS174 cells compared to total Peph extract

Treatment	Cell viability (%)
None	100 ± 3
Quercetin (Q)	100 ± 4
Rutin (R)	90 ± 1.3
(+)-Catechin (+C)	97 ± 4
(−)-Catechin (−C)	89 ± 3.9
Hyperin (H)	99 ± 3
Isoquercitrin (I)	91 ± 4
Chlorogenic acid (ChA)	93 ± 1
Cryptochlorogenic acid (CrA)	100 ± 4
Neochlorogenic acid (NeA)	94 ± 3.3
P-coumaric acid (PcA)	90 ± 1.97
Kaempferol (K)	100 ± 3.2
Kaempferol-3-*O*-glucoside (K3g)	100 ± 1.2
Kaempferol-3-*O*-rutinoside (K3r)	97 ± 2.4
Peph	26 ± 1.3

**Table 2:** Effect of binary combinations of Peph phenolic compounds on proliferation of LS174 cells

Viability (%)	Q	R	−C	+C	H	I	ChA	CrA	NeA	PcA	K	K3g	K3r
Q	–	79 ± 3	92 ± 2.9	89 ± 4.5	94 ± 9.1	87 ± 6.3	91 ± 2.7	80 ± 2.9	81 ± 1.7	93 ± 2.2	99 ± 4.7	94 ± 1.5	90 ± 4.2
R	79 ± 3	–	100 ± 9.1	78 ± 0.9	93 ± 1.8	84 ± 5.4	96 ± 7.4	76 ± 4.1	81 ± 1.8	75 ± 0.23	92 ± 4.2	89 ± 9.1	90 ± 0
−C	92 ± 2.9	100 ± 9.1	–	90 ± 8.9	93 ± 4.4	85 ± 0.14	79 ± 2.4	93 ± 5	89 ± 5.5	90 ± 7.9	92 ± 0.98	83 ± 8.4	93 ± 5.3
+C	89 ± 4.5	78 ± 0.9	90 ± 8.9	–	90 ± 2.4	83 ± 2.1	100 ± 1.7	82 ± 2.19	91 ± 0.28	89 ± 1.55	99 ± 7.3	100 ± 4.3	93 ± 4.45
H	94 ± 9.1	93 ± 1.8	93 ± 4.4	90 ± 2.4	–	90 ± 1.9	85 ± 2.1	87 ± 6.6	89 ± 1.5	92 ± 0.7	99 ± 4	84 ± 3.5	83 ± 8.2
I	87 ± 6.3	84 ± 5.4	85 ± 0.14	83 ± 2.1	90 ± 1.9	–	82 ± 4.2	78 ± 1.6	80 ± 2	78 ± 1.2	81 ± 1.1	98 ± 7.4	79 ± 5.4
ChA	91 ± 2.7	96 ± 7.4	79 ± 2.4	100 ± 1.7	85 ± 2.1	82 ± 4.2	–	80 ± 1.9	94 ± 7.1	84 ± 1.4	100 ± 2.7	85 ± 0.18	98 ± 0.9
CrA	80 ± 2.9	76 ± 4.1	93 ± 5	82 ± 2.19	87 ± 6.6	78 ± 1.6	80 ± 1.9	–	85 ± 1.7	92 ± 1.3	85 ± 4.8	85 ± 1.8	97 ± 1.1
NeA	81 ± 1.7	81 ± 1.8	89 ± 5.5	91 ± 0.28	89 ± 1.5	80 ± 2	94 ± 7.1	85 ± 1.7	–	83 ± 5.1	100 ± 1.5	100 ± 1.4	91 ± 4.1
PcA	93 ± 2.2	75 ± 0.23	90 ± 7.9	89 ± 1.55	92 ± 0.7	78 ± 1.2	84 ± 1.4	92 ± 1.3	83 ± 5.1	–	81 ± 0.19	84 ± 1.9	79 ± 3.7
K	99 ± 4.7	92 ± 4.2	92 ± 0.98	99 ± 7.3	99 ± 4	81 ± 1.1	100 ± 2.7	85 ± 4.8	100 ± 1.5	81 ± 0.19	–	83 ± 4.2	99 ± 2.8
K3g	94 ± 1.5	89 ± 9.1	83 ± 8.4	100 ± 4.3	84 ± 3.5	98 ± 7.4	85 ± 0.18	85 ± 1.8	100 ± 1.4	84 ± 1.9	83 ± 4.2	–	89 ± 3.2
K3r	90 ± 4.2	90 ± 0	93 ± 5.3	93 ± 4.45	83 ± 8.2	79 ± 5.4	97 ± 1.1	97 ± 1.1	91 ± 4.1	79 ± 3.7	99 ± 2.8	89 ± 3.2	–

### Quince peel polyphenols cause G1 phase cell cycle arrest and inhibit the expression of cell-cycle regulator cyclin D1 in LS174 cells

**Fig. 3:**
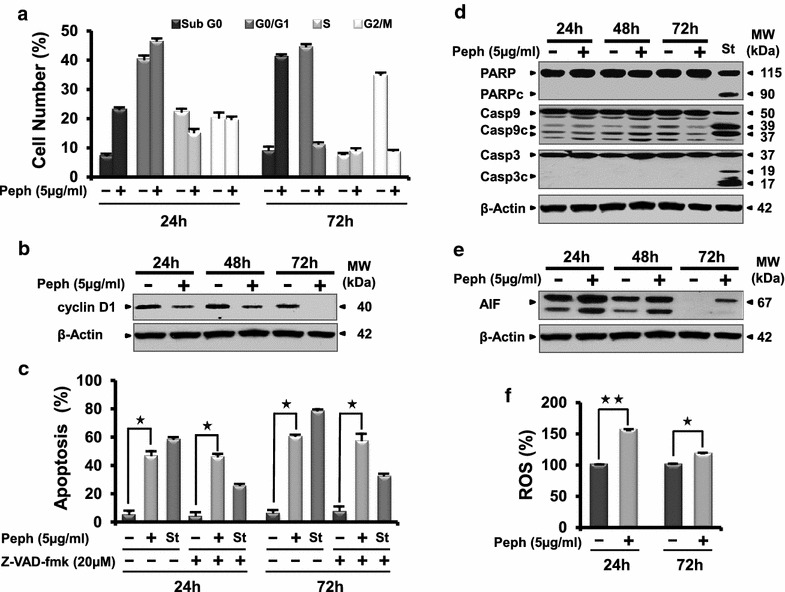
Quince peel polyphenolic extract induces cell cycle arrest, caspase-independent apoptosis and ROS generation in LS174 cells. **a** Histogram analysis of the cell cycle phases in mock- and treated-LS174 cells with 5 µg/ml of Peph for 24 and 72 h by flow cytometry using propidium iodide assay. **b** Quince peel polyphenolic extract down-regulates the expression level of cyclin D1 protein. Whole cell lysates (30 µg), from LS174 cells treated with Peph (5 µg/ml) or vehicle for 24–72 h, were resolved on SDS-PAGE gel and probed with the indicated antibody for western blot analysis. β-actin was used as a control for equal loading. **c** Flow cytometry analysis using annexin V/7-AAD staining of Z-VAD-FMK (20 µM)-pretreated LS174 cells cultured in the absence (control) and presence of 5 µg/ml of Peph extract for 24 and 72 h. Annexin V/7AAD double staining discriminates the live cells (Annexin V−/7AAD−; *bottom left* quadrant), early apoptotic cells (Annexin V+/7AAD−; *bottom right* quadrant), late apoptotic or necrotic cells (Annexin V+/7AAD+; *upper right* quadrant), and dead cells (Annexin V−/7AAD+; *upper left* quadrant). **d** Compared to the positive control (Staurosporine), western blot analysis showed the absence of caspase 3, 9 activities in LS174 cells after incubation for 72 h with Peph extract. **e** Induction of caspase-independent pathways mediated by the increase of apoptosis-inducing factor AIF proteins after treatment by quince Peph extract. **f** ROS production was measured with CMH2DCFDA staining after 24 and 72 h of treatment with 5 µg/ml of peel polyphenolic extract. Fluorescence was used to detect ROS as described in “Methods”. The data are representative of three independent experiments. Data are reported as the mean ± SE of three separate experiments (*p < 0.05)

A major event leading to carcinogenesis is the disruption of cell division cycle control through the dysregulation of cell cycle regulatory proteins [17]. In accordance with this, we explored the effect of Peph treatment on the cell cycle phase distribution of LS174 cells. As shown in Fig. 3a, treatment of the cells for 24 h with 5 µg/ml of Peph increased sub-G0 population (from 7 to 22.9 %) indicating apoptotic population. Accumulation of cells in G1 phase (from 40 to 46.37 %) was accompanied with a decrease in the S phase population (from 22 to 15 %). Within 72 h of treatment, Peph induced cell cycle perturbation and accumulation of 41 % of cells in sub-G0 phase. Since cyclin D1 protein is an important regulator of cell cycle progression from the G1-phase to the S-phase, we investigated its expression level during treatment of the cells with 5 µg/ml of Peph extract. The results showed that the expression level of cyclin D1 was reduced (Fig. 3b), suggesting its involvement in the Peph-induced LS174 cell cycle perturbation.

### Quince peel polyphenols induce a caspase-independent apoptosis of LS174 cells

The increase of the sub-G0 population after Peph treatment (Fig. 3a) along with the typical apoptotic morphological changes (nuclear fragmentation and membranes blebbing) observed by microscopy (data not shown) suggest that the decreased LS174 cell viability was a result of cell apoptosis. Flow cytometric analysis showed an increase of the percentage of annexin-V positive cells (43.7 %) after 24 h of treatment with 5 µg/ml of peel polyphenolic extract. The percentage of apoptotic cells increased to 54 % after 72 h of treatment, supporting the induction of cells death (Fig. 3c). To further explore the mechanism by which Peph extract induced cell apoptosis, we examined the caspases activity by western blotting. As showed in Fig. 3d, the peel polyphenols-induced apoptosis was not associated with the activation of caspase-3 and caspase-9. Moreover, caspase-3 substrate, PARP, was not cleaved in Peph-treated cells, suggesting that Peph induced a caspases-independent apoptosis of LS174 cells. To better verify this result, Z-VAD-fmk, a universal pan-caspase inhibitor, was added to the medium along with Peph. As shown in Fig. 3c, the caspase inhibitor did not reduce Peph-induced apoptosis while it diminished the cell death induced by the positive control, staurosporin (St). The apoptotic inducing factor (AIF), is considered as the main molecular effector and mediator of caspase-independent apoptosis [18]. Interestingly, we found that the treatment of the cells by the Peph extract increased the expression of AIF protein (Fig. 3e). These results suggest that the antiproliferative effect exerted by the quince peel phenolic extract passes through the induction of AIF, leading to caspase-independent apoptosis of the treated cells.

### Quince peel polyphenols induce ROS production

It is well established that the reactive oxygen species (ROS) could cause cell apoptosis [19]. We thus assessed the effect of Peph extract on the intracellular redox status of LS174 cells after 24 and 72 h of treatment. Pretreatment of LS174 cells with 5 µg/ml of Peph extract increased the level of ROS compared to mock-treated cells (Fig. 3f), suggesting their involvement in Peph-induced apoptosis of LS174 cells.

### Quince peel polyphenols-induced apoptosis is associated with the inhibition of NF-κB activity

**Fig. 4:**
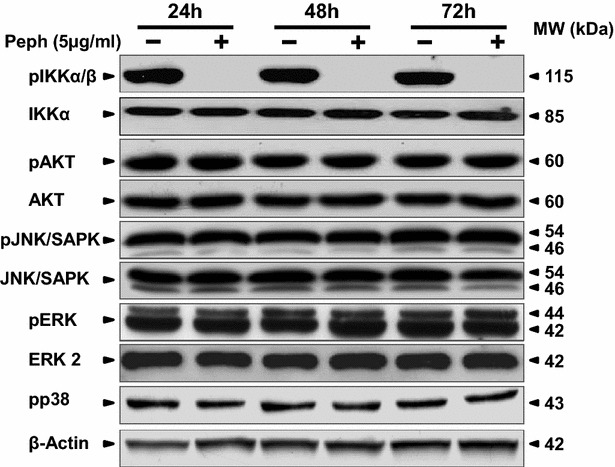
Quince peel polyphenolic extract blocks the activation of NF-κB. LS174 cells were treated with vehicle or with Peph (5 µg/ml) for 24, 48 and 72 h. Protein extracts (30 µg) prepared were analyzed by western blotting using the indicated antibodies. β-actin was used as a loading control. Data are representative of three independent experiments

To further characterize the mechanisms by which peel polyphenols inhibited cell growth, induced apoptosis and arrested cell cycle progression, we tested the involvement of MAPK (JNK, p38, and ERK_1/2_) cascades, PI3K/AKT and NF-κB signaling pathway that play critical role in colorectal cancer [20]. As shown in Fig. 4, the peel extract suppressed the NFκB activation as demonstrated by the inhibition of IkB kinase (IKK) phosphorylation (Fig. 4), suggesting the involvement of such pathway in Peph effects. However, we revealed no interference with the activity of the other studied kinases such as ERK_1/2_, p38 MAPK, SAPK/JNK and AKT in Peph-treated-LS174 (Fig. 4).

### Quince peel polyphenols inhibit the expression of the angiogenic effector VEGF-A

A high level of the potent angiogenic factor VEGF has been shown to be associated with colon cancer progression [21]. Since, VEGF expression is directly regulated by the NF-κB transcription factor [22], we assessed the effect of Peph extract-mediated inhibition of NF-κB on the angiogenic mediator VEGF-A.

**Fig. 5:**
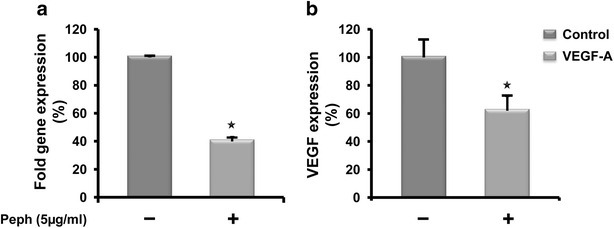
Peel polyphenolic extract inhibits VEGF-A expression in human colon cancer LS174 cells. LS174 cells were treated with vehicle or Peph (5 µg/ml) for 72 h. **a** The amount of VEGF-A mRNA transcript was quantified by real time PCR. The values are normalized to 36B4 and the control value was taken as 100 %. **b** Supernatants from LS174 cultured in the absence (vehicle) or presence of Peph (5 µg/ml) were collected and analyzed by Human VEGF specific ELISA. Results are reported as the mean ± SE of three independent experiments each run in triplicate (*p < 0.05). The data were corrected to the cell number

The levels of mRNA and proteins encoding for VEGF were examined by real-time qPCR and ELISA analysis in mock and Peph-treated LS174 cells. We found that VEGF-A expression was significantly reduced in Peph-treated cells at both the mRNA and protein levels (Fig. 5). This data suggest an additional potential anti-angiogenic activity of peel polyphenols through the inhibition of VEGF expression.

### Quince peel polyphenols markedly reduce tumor cell colony formation and potentiate the effect of 5-fluorouracil chemotherapy

**Fig. 6:**
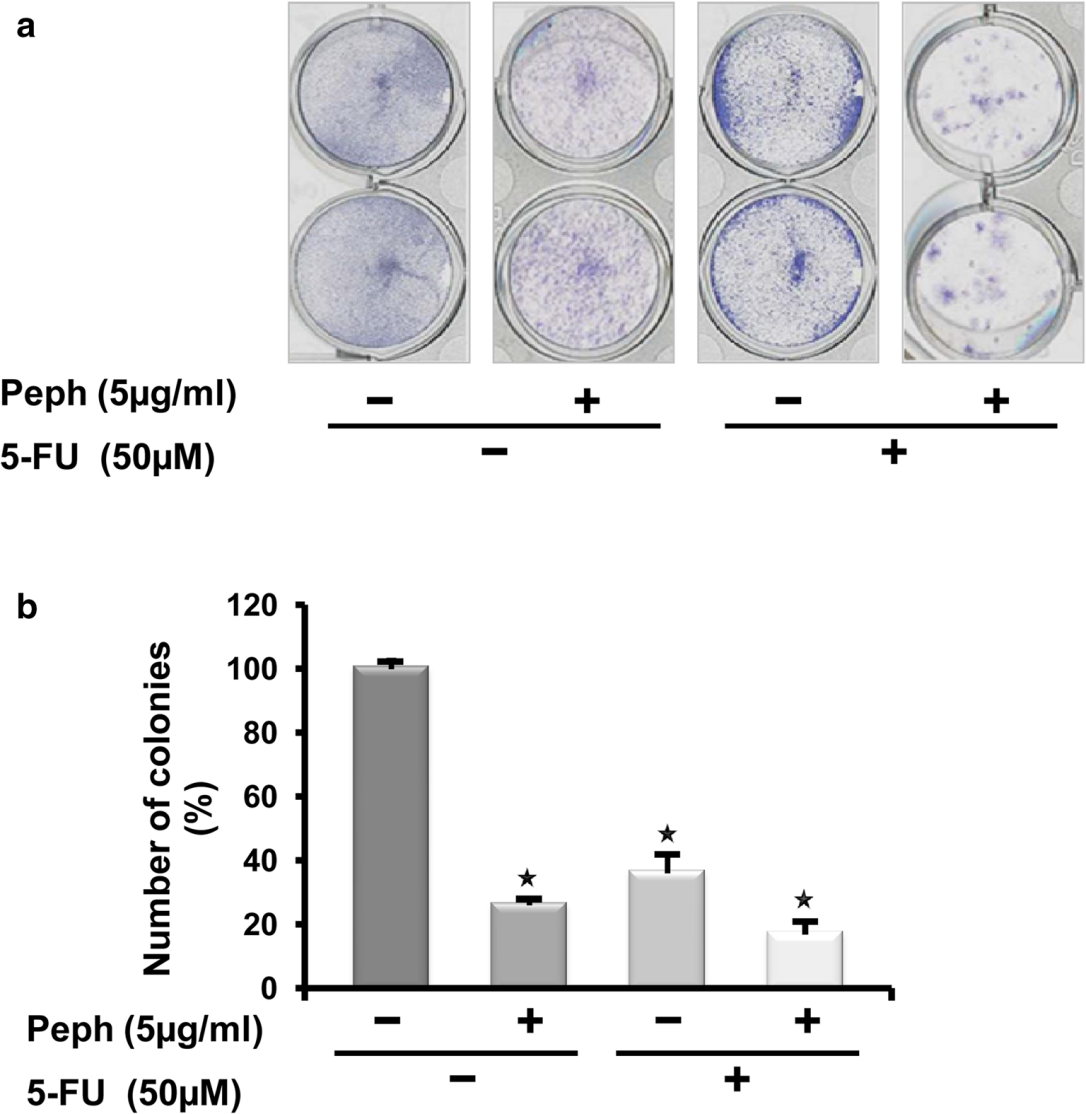
Quince peel polyphenols markedly reduce tumor cell colony formation and potentiates 5-fluorouracil efficacy. Peph effect on LS174 colony-forming capacity was measured using clonogenic survival assay. LS174 cells were treated with vehicle (control) or Peph (5 µg/ml) associated or not with 5-FU (50 µM) for 72 h. After removal of the medium, 2000 viable cells from each group were cultured in six-well plates for additional 10 days. Colonies were stained with crystal violet. Each assay was photographed (**a**) and the number of colonies was analyzed and scored by Image J quantification software. Results are expressed as the number of colony forming cells per well and normalized to control (vehicle) in percentage and represented as mean ± SE of three independent experiments, each performed at least in duplicate (**b**). *Asterisk* represents a significant difference (*p < 0.05) as compared to no treatment group

To further investigate the anti-tumoral effect of quince peel polyphenolic extract and to test its long-term effect, we analyzed the impact of such extract on cancer colony formation along with its effect when combined to 5-FU, the conventional drug used in chemotherapy of epithelial solid tumors. We first found that treatment of cells with Peph extract alone had similar anti-tumor growth effect as the one exhibited by 5-FU, as shown by the decrease of viable colonies compared to non-treated cells (Fig. 6). Combined treatment of 5-FU with Peph extract caused more decrease in colony formation of cancer cells compared to 5-FU alone. These results showed that 5 µg/ml of Peph extract produced a strong anti-tumor activity when combined with 50 µM of 5-FU, indicating that Peph and 5-FU has synergistic anti-tumor effects on LS174 cells.

## Discussion

We have previously reported a potent anti-inflammatory effect of quince (*Cydonia oblonga* Miller) peel polyphenolic extract in LPS-stimulated human THP-1-derived macrophages [15]. Accumulating evidence shows that chronic inflammation is an important etiologic factor in the development of colorectal cancer [23] and several studies have shown that dietary polyphenols with anti-inflammatory properties are effective against different types of cancer [24]. Our aim was to investigate the anti-colon cancer effect of a non toxic natural extract from quince as an alternative to treat and prevent the growth and development of colorectal cancer. In this work we show, for the first time, that peel polyphenolic extract of *Cydonia* *oblonga* Miller goes further than an antiproliferative agent in colon cancer cells, displaying pro-apoptotic and anti-angiogenic activities along with the capacity to enhance chemotherapeutic effectiveness of 5-fluorouracil.

We first found that the Tunisian quince (*Cydonia oblonga* Miller) aqueous acetone extract presented a dose-dependent inhibition effect on the viability of colon LS174 adenocarcinoma cells, while no effect was observed in the non-tumorigenic cells. Our data indicate that the peel polyphenolic extract inhibited the proliferation of human LS174 colon cancer cells without any toxic effect, as assessed with LDH assay. This correlates with previous work done by Carvalho et al. [16] that reported an inhibition effect of the methanolic extract of *Cydonia oblonga* Miller on the proliferation of colon Caco-2 cancer cells. We have further found that quince peel polyphenols (Peph) presented higher antiproliferative effect than those extracted from the pulp (Puph). This might be explained by the difference in the phenolic composition of each extract. Indeed, according to the results we previously reported [13, 15], total phenolic content of the pulp and peel parts of quince (*Cydonia* *oblonga* Miller) extracts ranged from 37 to 47 and 105 to 157 mg/100 g of fresh weight, respectively. Phenolic components level in the peel fraction is three times higher than the one of the pulp. Phytochemical investigations led to the identification of thirteen phenolic compounds in quince Peph extract [13]. Previous studies highlighted the anti-cancer properties of the isolated phenolic molecules in tumor cells. Chlorogenic acid, a phenolic molecule found in the quince peel polyphenolic extract, has been reported to induce apoptotic cell death in U937 leukemia cells [25]. Rutin, another major component of the peel quince polyphenolic extract (36 %), has been also reported to exert antitumoral effects in human colon cancer cells [26]. Moreover, quercetin and kaempferol, additional members of the quince peel polyphenols, effectively inhibits pancreatic tumour growth in vitro and in vivo via an increase in apoptosis [27]. All the remaining components of the peel quince polyphenolic extract like catechin and procyanidin have revealed promising chemopreventive and/or anticancer efficacy in several cell cultures and animal models [28]. The use of commercially purified compounds based on their concentration and percentage in 5 µg/ml of Peph extract have indicated that the antiproliferative effect of Peph involves total polyphenolic compounds (Table 1). However, in the context of our study, we still do not know which combination of compounds from the peel phenolic extract is behind the antiproliferative effect (Table 2; Additional file 1: Figure S1). Our data suggest that a synergistic effect between different molecules may have contributed to the antiproliferative effect of the Peph extract, on LS174 adenocarcinoma cells. This supports the fact that there are usually more benefits to use a whole natural plant extract, with different pharmacologically active phytochemicals, than a single isolated compound. Indeed, total compounds within Peph extract could inhibit any toxic effects of one compound alone and have many different intracellular targets, which may act in a synergistic way to enhance specific activity. Additionally, the presence of multiple components may possibly decrease the chances of developing chemoresistance [29]. Moreover, natural extracts like quince can be administered orally to patients, as a safe mode of administration. Many works have focused on the bioavailability and polyphenols bioefficacy in humans [30]. Polyphenol concentrations in the colon can reach several hundred micromoles per liter, and together with a few carotenoids, polyphenols constitute the only dietary present in the colon, because vitamins C and E are absorbed in the upper segments of the intestine [30, 31].

Based on the fact that the total peel polyphenolic extract of *Cydonia oblonga* Miller is endowed with the potent antiproliferative activity against human colon carcinoma LS174 cells along with the cited biological activities of its compounds, we tried to uncover the anticancer mechanisms of quince polyphenolic extract. Flow cytometry analysis showed an increase in the percentage of annexin-V positive cells when treated with quince Peph extract. Such apoptotic feature has been reported to be frequently associated with increased caspases activity [32]. However, treatment with a pan-caspase inhibitor (z-VAD-fmk) did not block the apoptotic cell death in Peph-treated LS174 cells. Furthermore, the Peph-induced apoptotic effect was not accompanied by processing of procaspase-3,-9, and PARP confirming that quince peel polyphenols induced LS174 cell apoptosis through a caspase-independent pathway. Endonuclease G (Endo G) and AIF are key factors, released by the mitochondria, that are able to induce a caspase-independent apoptosis [33]. Interestingly, the quince Peph extract-induced apoptosis of LS174 cells was associated with an increase of AIF level but no effect was observed concerning Endo G expression level (Data not shown). Our result is in agreement with several studies reporting that various chemotherapeutic agents induced AIF-mediated apoptosis in a large array of colon cancer cell lines [34]. Our data also correlate with a previous work done by Wang et al. [35] who reported that Berberine, an isoquinoline alkaloid derived from plants, induces caspase-independent cell death in colon tumor cells through activation of AIF. This latter is also a Janus protein that directly regulates ROS production in the mitochondria [36]. In accordance with this, we investigated the potential of quince peel polyphenolic extract to modulate the intracellular amount of ROS. Interestingly, we observed an increase of ROS level after 24 and 72 h of treatment with Peph extract. This result suggests that the pro-apoptotic effect of Peph extract may, at least in part, be caused by ROS production. Our data is in agreement with previous studies that reported a high level of ROS production in anti-cancer therapeutic strategies based on the activation of the AIF caspase-independent cell death [36].

Colon cancer has been also shown to be associated with an overexpression of growth promoting cell cycle regulators such as cyclin D1 [37]. An aberrant accumulation of this regulator occurs in about one-third of colorectal cancer [38]. Interestingly, we found that Peph induced the downregulation of cyclin D1. This data suggests that the antiproliferative effect of Peph phenolic extract pass through the inhibition of cyclin D1 expression, causing then the cell cycle arrest before the engagement of tumor cells into an apoptotic cell death. Multiple signaling pathways such as Wnt/β-catenin, MAPK, PI3 K, JAKs/STAT3 and NF-κB signaling pathways are implicated in colorectal cancer development [7, 39]. We have then explored the possible modulation, by Peph extract, of NF-κB, PI3 K/AKT and MAPK cascades pathways including ERK, JNK, and p38MAPKs. Analysis of the indicated pathways by western blot demonstrated that MAPK activity was not involved in the antiproliferative and apoptotic effect of Peph extract. These data are consistent with those reported by Cho et al. [40] that found no involvement of the MAPK cascades in the caspase-independent apoptosis of A172 glioma cells induced by 15d-PGJ2. Our results also demonstrate that the PI3 K/AKT survival pathway was not affected by Peph extract. However, Peph extract treatment of the LS174 cells inhibited the NF-κB signaling pathway which is recognized as a key player in the initiation and propagation of CRC [11]. This data are also in accordance with a most recent review where authors reported that inhibition of the NF-κB signaling contributes to the anticancer effects of some therapeutic drugs to prevent colon cancer metastasis [41]. These data also correlate with our previous work using THP-1-derived macrophages. Indeed, we reported that one way by which quince Peph extract inhibits LPS-induced inflammation, is through the blockade of NF-κB activation [15]. NFκB orchestrates the expression of inflammatory cytokines, adhesion molecules and angiogenic factors [42]. Moreover, it has been shown that the inhibition of angiogenesis is mediated by the blocking of NFκB activation [22]. VEGF-A, the most important regulator of tumor angiogenesis, is the first-choice target of anti-angiogenic therapies [43]. Clinical studies on patients with CRC suggest that VEGF-A expression is significantly higher in metastatic tumors than in non metastatic tumors, and the increased VEGF-A expression is related to the worse prognosis [41]. In accordance with this, we have then investigated the potential of quince Peph extract to modulate the expression and secretion of VEGF-A. Interestingly, we found that quince polyphenols inhibited VEGF-A gene expression, as assessed by Real-Time quantitative PCR. We have also found that such effect was associated with a down-regulation of VEGF-A secretion. These data suggest that the inhibition effect, exerted by Peph extract on NFκB pathway caused the down-regulation of VEGF expression which may lead to the blocking of tumor-induced angiogenesis.

An understanding of the angiogenic pathways has progressed for the development of colorectal treatment [44]. Bevacizumab is a humanized monoclonal antibody which exerts its effect by inhibiting the effect of VEGF-A thus inhibiting it’s binding to the VEGF receptor and prevents angiogenesis [45]. However, the indicated drugs suffer from being expensive for a larger use, besides they present several harmful side effects. Our results suggest that quince peel polyphenolic extract could represent an alternative since it has chemopreventive and chemotherapeutic properties through its dual inhibitory effect of both colon cancer cell survival/proliferation and angiogenesis promoting molecules. Recent studies reported the promising possibilities of use of dietary compounds to sensitize tumors to chemotherapeutics agents [46]. Combinations of chemotherapeutical agents with secondary metabolites that have different modes of action can decrease the systemic toxicity because they allow lowering the concentrations of the most toxic therapeutic drug [47]. We have then assessed the effect of quince Peph extract in combination to 5-Fluorouracil (5-FU), a classical anticancer drug recommended for first-line treatment in CRC [48]. Our result demonstrate that the combined treatment of 5-FU with Peph extract caused a significant decrease in colony formation of colon cancer cells compared to 5-FU alone. This finding suggest that quince Peph extract may improve the therapeutic effects of 5-Fluorouracil.

In the light of our study, quince peel polyphenolic extract, a natural product appears to be a promising potential anti-tumoral agent. Actually, natural products are the source of many drugs in cancer therapy and approximately 75 % of the approved anticancer therapies have been derived from natural products [49].

## Conclusion

We highlighted an interesting pharmacological effect for peel polyphenolic extract of *Cydonia oblonga* Miller. We demonstrated for the first time that this extract induces proliferation arrest and apoptosis of colon cancer LS174 cells and that such effect is at least partially mediated by inhibition of NF-κB activation. This extract also lowered VEGF-A expression and secretion by tumour cells which may lead to the inhibition of tumor-induced angiogenesis. We finally found that quince peel polyphenolic extract enhances the therapeutic activity of 5-Fluorouracil in colon cancer LS174 cells.

It is worth to note that in our work this effect was due to total components of the phenolic extract that have acted synergistically to generate the potent anti-tumor effect. However, we must underline the importance of using the totem of quince peel polyphenols to benefit of its total bioactive compounds. Indeed, the studied extract is a non toxic natural food product that contains a mixture of anti-tumor bioactive molecules that can potentially be used alone or combined to the conventional chemotherapeutic agents used to treat colon cancer. Work is in progress to generate a 5-Fu-resistant LS174 cell line model to study the effect of peel polyphenolic extract and potential synergistic interactions of its compounds in this experimental system. Further investigation is also needed to better verify if the in vitro anti-tumour effect of quince polyphenols extract in human colon adenocarcinoma LS174 cells can be extended to the in vivo setting. Detailed pharmacokinetic and toxicological studies with experimental animals are required before such a strategy can be translated into clinical practice.

## Methods

### Quince polyphenols extraction

Quince polyphenols preparations along with identification and quantification of their compounds using high-performance liquid chromatography with diode-array detection (HPLC–DAD) coupled on line to a mass spectrometer (MS) were performed as previously reported [13, 15]. The thirteen identified pure compounds present in the quince peel polyphenolic extract (Peph) (Quercetin (Q), Rutin (R), (+)-Catechin (+C), (−)-Catechin (−C), Hyperin (H), Isoquercitrin (I), Chlorogenic acid (ChA), Cryptochlorogenic acid (CrA), Neochlorogenic acid (NeA), *p*-coumaric acid (PcA), Kaempferol (K), Kaempferol-3-*O*-glucoside(K3g) and Kaempferol-3-*O*-rutinoside (K3r)) were purchased from Sigma (Sigma, Aldrich).

### Cell culture and treatment

Human colon adenocarcinoma LS174 cell line (CL-188), and non-tumourigenic cells human embryonic kidney HEK293 (CRL1573) and mouse embryonic fibroblasts NIH3T3 (CRL-1658) were obtained from American Type Culture Collection (**ATCC,** Manassas, VA). The cells were cultured in DMEM (Dulbecco’s Modified Eagle’s Medium) supplemented with 10 % fetal bovine serum (FBS; GIBCO) and 50 U/ml penicillin and 50 µg/ml streptomycin. Cells were seeded with an adequate cell density and treated with different concentrations of quince polyphenolic extract in triplicate and incubated for 24, 48 and 72 h.

### Measurement of cell viability

Cell viability was determined with colorimetric MTT (3-(4,5-dimethylthiazol-2yl)-2,5-diphenyl tetrazolium bromide) assay. The non-tumourigenic HEK293 and NIH3T3 cells and human colon adenocarcinoma LS174 cells were seeded in 96-well plates (1000 cells/well). After 24 h, the cells were treated with serial concentrations of quince polyphenolic extracts (1, 2.5, 5, 10, 15 and 20 µg/ml) or pure phenolic compounds at equivalent concentrations to that present in 5 µg/ml of Peph for various periods (24, 48 and 72 h). In this assay, a colorless tetrazolium salt (MTT) is cleaved and converted to blue formazan by the mitochondrial dehydrogenase in living cells. Thereafter, 50 µl of a MTT solution (1 mg/ml final) was added into each 96-well plate, and the cells were incubated for a further 3 h at 37 °C, 100 µl of DMSO was added to each well to dissolve the blue formazan. The optical density (OD) at 540 nm was measured with a microplate reader (MULTISKAN, Labsystems). The cell viability was expressed as percentage of the viable cell number in treated cells relative to mock-treated cells (control). Cell viability was calculated using the following formula: % cell viability = OD_test_/OD_control_ × 100.

### LDH release assay

Cellular membrane integrity was monitored by the permeability assay based on the release of lactate dehydrogenase (LDH) into the media. LDH release from non-tumourigenic cells HEK293, NIH 3T3 and Human colon adenocarcinoma LS174 cells was determined by using the LDH Cytotoxicity Detection Kit-PLUS test (Roche Applied Science, Mannheim, Germany) according to the manufacturer’s protocol. Briefly, cells were seeded in 12-well plates (10^5^cells/well) and cultured for 72 h with different concentrations (5, 10 and 20 µg/ml) of quince polyphenolic extracts (Peph and Puph). The percentage release of LDH was calculated from the treated cells by comparing it with the maximum release obtained by 1 % Triton X-100 treatment (positive control) and the spontaneous LDH release (mock-treated cells considered as a negative control) as follows: Cytotoxicity (%) = (experimental value–negative control)/(positive control–negative control) × 100.

### Cell cycle phase distribution analysis

LS 174 cells were seeded into six-well plates at a density of 2 × 10^5^ cells/ml, grown for 24 h and then exposed for 24 and 72 h to 5 µg/ml of quince peel polyphenolic extract (Peph). Thereafter, cells were collected by centrifugation at 1000 rpm, washed twice with 1X ice-cold PBS containing 2 % bovine albumin serum (BSA) (Sigma) and resuspended in Hypertonic solution (20 mM HEPES, pH 7.2; 0.16 M NaCl; 1 mM EGTA; 0.05 % Triton X-100). After incubation at 4 °C, cells were washed, treated with propidium iodide (PI)/RNase staining solution (Cell Signaling Technology; Danvers, MA) and incubated for at least 30 min in the dark at 37 °C. Cell cycle distribution profiles were analyzed on a Becton–Dickinson FACScanto II flow cytometer and further analyzed with BD FACSDiva 6 software (Becton–Dickinson). The PI fluorescence signal at FL2-A peak versus counts was used to determine cell cycle distribution and the data were analyzed using the Modfit software. The percentage of cells in SubG0, G0/G_1_, S and G_2_/M was determined.

### Apoptosis detection

Detection and quantification of apoptosis was performed by the analysis of phosphatidylserine on the outer leaflet of apoptotic cell membranes using annexin V/PE apoptosis detection kit (BD-Pharmingen) according to the manufacturer’s protocol. Approximately 10^5^ cells, treated with 5 µg/ml of Peph for 24 and 72 h or vehicle as a negative control were collected by centrifugation, and washed with PBS (1×). For caspase inhibitor activity assay, cells were pre-incubated with a pan-caspase inhibitor, Z-VAD-FMK (20 µM) (BD Pharmingen) for 2 h before Peph-treatment. LS174 cells were resuspended in 100 μl of binding buffer (1×) before addition of 4 μl of Annexin V conjugated to phycoerythrin (PE) and 4 µl of 7-AAD. The cell suspension was incubated in the dark rapidly for 15 min. Stained cells were analyzed on a Becton–Dickinson FACScanto II flow cytometer and further analyzed with BD FACSDiva 6 software (Becton–Dickinson). Cell death was quantitatively evaluated by measuring the proportion of annexin V-positive cells, regardless of their staining for 7-AAD in order to include both apoptotic and necrotic cell death. Values are given in percent of total cell number. Percentage of apoptotic cells (%) was calculated as follows: early apoptotic cells (%) + late apoptotic cells (%).

### Measurement of reactive oxygen species (ROS)

The intracellular level of ROS was determined using a cell-permeable fluorogenic probe, CMH2DCF-DA (life technologies, Oregon, USA). This molecule passively diffuses into cells, where its acetate groups are cleaved by intracellular esterases and its thiol-reactive chloromethyl group reacts with intracellular glutathione and other thiols. Subsequent oxidation yields a fluorescent adduct in the presence of ROS. LS174 cells were seeded in 96-well plates (2000 cells/well) and treated with 5 µg/ml of Peph for 24 and 72 h. Cells were washed with PBS (1×), resuspended in HBSS (GIBCO) and incubated with CMH2DCFDA (10 µM) at 37 °C for 30 min in dark. Fluorescence was detected with excitation and emission wavelength at 492 and 517 nm respectively.

### Western blot analysis

At various times (24, 48 and 72 h) after 5 µg/ml of peel polyphenols treatment, the LS174 cells were collected and lysed at room temperature with 100 µl of Laemmli buffer (1×). Protein content of the cell lysates was quantified using the BCA method (Bicinchoninic Acid Protein Assay kit, Sigma). Equal amounts of protein (30 µg/sample) were separated electrophoretically by 10 % SDS-PAGE and blotted onto PVDF membranes (Immobilon-Millipore). The blots were probed with primary antibodies and incubated with a horseradish peroxidase-conjugated anti-IgG in a blocking buffer for 1 h. Primary antibodies anti-phospho-AKT, anti-AKT, anti-ERK_2_, anti-phospho-JNK/SAPK, anti-JNK/SAPK, anti-phospho-p38, anti-phospho-IKKα/β, anti-IKKα, anti-PARP, anti-caspase 9, anti-caspase 3, anti-AIF, anti-Cyclin D1 and loading control anti-actin were from cell Signaling Technology (Danvers, MA, USA). Anti-phospho ERKs were from Sigma-Aldrich (L’Isle d’Abeau, Chesnes, France), and anti-horseradish peroxidase-conjugated anti-mouse and anti-rabbit antibodies were from Promega (Madison, WI). After washing, the blots were developed with enhanced chemiluminescence (ECL) (Millipore) and exposed to X-ray film.

### Real time quantitative RT-PCR

To evaluate the expression patterns of up-regulated or down-regulated genes after 5 µg/ml of peel polyphenols (Peph) treatment, the selected genes were chosen for further analysis using real-time quantitative reverse transcription-PCR (RTQ-RT-PCR) in a 96-well format. Real-time PCR measures of VEGF-A cDNA expression were obtained using a Light Cycler with a Fast Start DNA Master Mix SYBR Green. The 2^[−ΔΔC (T)]^ method was used to calculate the relative expression of gene, as previously described [50].

### Determination of cytokine concentrations

Cell culture supernatants from LS174 cells treated with 5 µg/ml of peel polyphenolic extract (Peph) for 72 h were collected centrifuged at 10,000 rpm for 5 min and the cells were counted. Determination of the VEGF-A concentrations was carried out using an ELISA kit Quantikine human VEGF Immunoassay (Pierce, Rockford, USA) following the manufacturer’s guidelines and normalized to cell number.

### Colony formation assay

For clonogenic assays, LS174 cells were plated in six-well plates (2 × 10^5^cells/well) and treated the day after with 5 µg/ml of peel polyphenolic extract alone or in combination with 50 μM of 5-Fluorouracil for a period of 72 h. After removal of the medium containing antitumor drugs, cells were trypsinized and plated at low density (2000 cells per six-well plate). Cells were then cultivated for 10 days. Colonies were stained with crystal violet and clones for each condition were scored by Image J quantification software. Results are expressed as the number of colony forming cells per well and normalized to control (vehicle) in percentage (considered to represent 100 %).

### Statistical analysis

Data from individual experiments are expressed as mean ± SE. Differences between means were evaluated using Student’s *t* test. Differences with *p* values of less than 0.05 were considered statistically significant.

## Additional file

**Additional file: Figure S1**. The effect of combination of Peph phenolic compounds on proliferation of LS174 cells compared to total Peph extract. Human colon adenocarcinoma LS174 cells were treated for 72 h with different combinations (**A**. 3 combinations, **B**. 4 combinations, **C**. 5 combinations) of Peph phenolic compounds. All compounds where tested at equivalent concentrations to that present in 5 μg/ml of the total peel polyphenolic extract. Cell viability was determined by MTT assay.

## References

[1] Araújo JR, Gonçalves P, Martel F (2011). Chemopreventive effect of dietary polyphenols in colorectal cancer cell lines. Nutr Res.

[2] Pericleous M, Mandair D, Caplin ME (2013). Diet and supplements and their impact on colorectal cancer. J Gastrointest Oncol.

[3] Lam M, Carmichael AR, Griffiths HR (2012). An aqueous extract of *Fagonia cretica* induces DNA damage, cell cycle arrest and apoptosis in breast cancer cells via FOXO3a and p53 expression. PLoS ONE.

[4] Lecci RM, Logrieco A, Leone A (2014). Pro-oxidative action of polyphenols as action mechanism for their pro-apoptotic activity. Anticancer Agents Med Chem.

[5] Li G, Thomas S, Johnson JJ (2013). Polyphenols from the mangosteen (*Garcinia mangostana*) fruit for breast and prostate cancer. Front Pharmacol.

[6] Khan HY, Zubair H, Ullah MF, Ahmad A, Hadi SM (2012). A prooxidant mechanism for the anticancer and chemopreventive properties of plant polyphenols. Curr Drug Targets.

[7] Wu WK, Wang XJ, Cheng AS, Luo MX, Ng SS, To KF (2013). Dysregulation and crosstalk of cellular signaling pathways in colon carcinogenesis. Crit Rev Oncol Hematol.

[8] Kim SW, Schifano M, Oleksyn D, Jordan CT, Ryan D, Insel R (2014). Protein kinase C-associated kinase regulates NF-κB activation through inducing IKK activation. Int J Oncol.

[9] Tripathi V, Popescu NC, Zimonjic DB (2014). DLC1 suppresses NF-κB activity in prostate cancer cells due to its stabilizing effect on adherens junctions. Springerplus.

[10] Aggarwal BB, Shishodia S (2006). Molecular targets of dietary agents for prevention and therapy of cancer. Biochem Pharmacol.

[11] Vaiopoulos AG, Athanasoula KCh, Papavassiliou AG (2013). NF-κB in colorectal cancer. J Mol Med.

[12] Khan MK, Ansari IA, Khan MS (2013). Dietary phytochemicals as potent chemotherapeutic agents against breast cancer: inhibition of NF-κB pathway via molecular interactions in rel homology domain of its precursor protein p105. Pharmacogn Mag.

[13] Fattouch S, Caboni P, Coroneo V, Tuberoso CI, Angioni A, Dessi S (2007). Antimicrobial activity of Tunisian quince (*Cydonia oblonga* Miller) pulp and peel polyphenolic extracts. J Agric Food Chem.

[14] Magalhães AS, Silva BM, Pereira JA, Andrade PB, Valentão P (2009). Protective effect of quince (*Cydonia oblonga* Miller) fruit against oxidative hemolysis of human erythrocytes. Food Chem Toxicol.

[15] Essafi-Benkhadir K, Refai A, Riahi I, Fattouch S, Karoui H, Essafi M (2012). Quince (*Cydonia oblonga* Miller) peel polyphenols modulate LPS-induced inflammation in human THP-1-derived macrophages through NF-κB, p38MAPK and Akt inhibition. Biochem Biophys Res Commun.

[16] Carvalho M, Silva BM, Silva R, Valentão P, Andrade PB, Bastos ML (2010). First report on *Cydonia oblonga* Miller anticancer potential: differential antiproliferative effect against human kidney and colon cancer cells. J Agric Food Chem.

[17] Malumbres M (2012). Cell cycle-based therapies move forward. Cancer Cell.

[18] Donovan M, Cotter TG (2004). Control of mitochondrial integrity by Bcl-2 family members and caspase-independent cell death. Biochim Biophys Acta.

[19] Sreevalsan S, Safe S (2013). Reactive oxygen species and colorectal cancer. Curr Colorectal Cancer Rep.

[20] Gunda V, Bucur O, Varnau J, Vanden Borre P, Bernasconi MJ, Khosravi-Far R (2014). Blocks to thyroid cancer cell apoptosis can be overcome by inhibition of the MAPK and PI3K/AKT pathways. Cell Death Dis.

[21] Sun W (2012). Angiogenesis in metastatic colorectal cancer and the benefits of targeted therapy. J Hematol Oncol.

[22] Zhang J, Peng B (2007). In vitro angiogenesis and expression of nuclear factor kappaB and VEGF in high and low metastasis cell lines of salivary gland Adenoid Cystic Carcinoma. BMC Cancer.

[23] Yashiro M (2015). Molecular alterations of colorectal cancer with inflammatory bowel disease. Dig Dis Sci.

[24] Al-Halabi R, Bou Chedid M, Abou Merhi R, El-Hajj H, Zahr H, Schneider-Stock R (2011). Gallotannin inhibits NFĸB signaling and growth of human colon cancer xenografts. Cancer Biol Ther.

[25] Yang JS, Liu CW, Ma YS, Weng SW, Tang NY, Wu SH (2012). Chlorogenic acid induces apoptotic cell death in U937 leukemia cells through caspase- and mitochondria-dependent pathways. In Vivo.

[26] Alonso-Castro AJ, Domínguez F, García-Carrancá A (2013). Rutin exerts antitumor effects on nude mice bearing SW480 tumor. Arch Med Res.

[27] Vuong QV, Hirun S, Phillips PA, Chuen TL, Bowyer MC, Goldsmith CD (2014). Fruit-derived phenolic compounds and pancreatic cancer: perspectives from Australian native fruits. J Ethnopharmacol.

[28] Dinicola S, Cucina A, Pasqualato A, D’Anselmi F, Proietti S, Lisi E (2012). Antiproliferative and apoptotic effects triggered by grape seed extract (GSE) versus epigallocatechin and procyanidins on colon cancer cell lines. Int J Mol Sci.

[29] Foster BC, Arnason JT, Briggs CJ (2005). Natural health products and drug disposition. Annu Rev Pharmacol Toxicol.

[30] Scalbert A, Williamson G (2000). Dietary intake and bioavailability of polyphenols. J Nutr.

[31] Scalbert A, Morand C, Manach C, Rémésy C (2002). Absorption and metabolism of polyphenols in the gut and impact on health. Biomed Pharmacother.

[32] D’Archivio M, Santangelo C, Scazzocchio B, Varì R, Filesi C, Masella R (2008). Modulatory effects of polyphenols on apoptosis induction: relevance for cancer prevention. Int J Mol Sci.

[33] Portt L, Norman G, Clapp C, Greenwood M, Greenwood MT (2011). Anti-apoptosis and cell survival: a review. Biochim Biophys Acta.

[34] Millan A, Huerta S (2009). Apoptosis-inducing factor and colon cancer. J Surg Res.

[35] Wang L, Liu L, Shi Y, Cao H, Chaturvedi R, Calcutt MW (2012). Berberine induces caspase-independent cell death in colon tumor cells through activation of apoptosis-inducing factor. PLoS ONE.

[36] Delavallée L, Cabon L, Galán-Malo P, Lorenzo HK, Susin SA (2011). AIF-mediated caspase-independent necroptosis: a new chance for targeted therapeutics. IUBMB Life.

[37] Mao Y, Li Z, Lou C, Zhang Y (2011). Expression of phosphorylated Stat5 predicts expression of cyclin D1 and correlates with poor prognosis of colonic adenocarcinoma. Int J Colorectal Dis.

[38] Li Y, Wei J, Xu C, Zhao Z, You T (2014). Prognostic significance of cyclin D1 expression in colorectal cancer: a meta-analysis of observational studies. PLoS ONE.

[39] Centuori SM, Martinez JD (2014). Differential regulation of EGFR-MAPK signaling by deoxycholic acid (DCA) and ursodeoxycholic acid (UDCA) in colon cancer. Dig Dis Sci.

[40] Cho WH, Choi CH, Park JY, Kang SK, Kim YK (2006). 15-deoxy-(Delta12,14)-prostaglandin J2 (15d-PGJ2) induces cell death through caspase-independent mechanism in A172 human glioma cells. Neurochem Res.

[41] Liu X, Ji Q, Fan Z, Li Q (2015). Cellular signaling pathways implicated in metastasis of colorectal cancer and the associated targeted agents. Future Oncol..

[42] Del Prete A, Allavena P, Santoro G, Fumarulo R, Corsi MM, Mantovani A (2011). Molecular pathways in cancer-related inflammation. Biochem Med.

[43] Bellou S, Karali E, Bagli E, Al-Maharik N, Morbidelli L, Ziche M (2012). The isoflavone metabolite 6-methoxyequol inhibits angiogenesis and suppresses tumor growth. Mol Cancer.

[44] Mathonnet M, Perraud A, Christou N, Akil H, Melin C, Battu S (2014). Hallmarks in colorectal cancer: angiogenesis and cancer stem-like cells. World J Gastroenterol.

[45] Yaffee P, Osipov A, Tan C, Tuli R, Hendifar A (2015). Review of systemic therapies for locally advanced and metastatic rectal cancer. J Gastrointest Oncol.

[46] Díaz-Chávez J, Fonseca-Sánchez MA, Arechaga-Ocampo E, Flores-Pérez A, Palacios-Rodríguez Y, Domínguez-Gómez G (2013). Proteomic profiling reveals that resveratrol inhibits HSP27 expression and sensitizes breast cancer cells to doxorubicin therapy. PLoS ONE.

[47] El-Readi MZ, Hamdan D, Farrag N, El-Shazly A, Wink M (2010). Inhibition of P-glycoprotein activity by limonin and other secondary metabolites from Citrus species in human colon and leukaemia cell lines. Eur J Pharmacol.

[48] Webber EM, Kauffman TL, O’Connor E, Goddard KA (2015). Systematic review of the predictive effect of MSI status in colorectal cancer patients undergoing 5FU-based chemotherapy. BMC Cancer.

[49] Davidson D, Amrein L, Panasci L, Aloyz R (2013). Small molecules, inhibitors of DNA-PK, Targeting DNA repair, and beyond. Front Pharmacol.

[50] Livak KJ, Schmittgen TD (2001). Analysis of relative gene expression data using real-time quantitative PCR and the 2(−Delta Delta C(T)) Method. Methods.

