# Supplementary material for: Quince peel polyphenolic extract blocks human colon adenocarcinoma LS174 cell growth and potentiates 5-fluorouracil efficacy
Source: Cancer Cell Int. 2016 Feb 2;16:1. doi: 10.1186/s12935-016-0276-7 (PMC4736700; doi:10.1186/s12935-016-0276-7)
Supplement: Supplementary file 1 — Additional file: Figure S1. The effect of combination of Peph phenolic compounds on proliferation of LS174 cells compared to total Peph extract. Human colon adenocarcinoma LS174 cells were treated for 72 h with different combinations (A. 3 combinations, B. 4 combinations, C. 5 combinations) of Peph phenolic compounds. All compounds where tested at equivalent concentrations to that present in 5 μg/ml of the total peel polyphenolic extract. Cell viability was determined by MTT assay. [file 12935_2016_276_MOESM1_ESM.pdf]

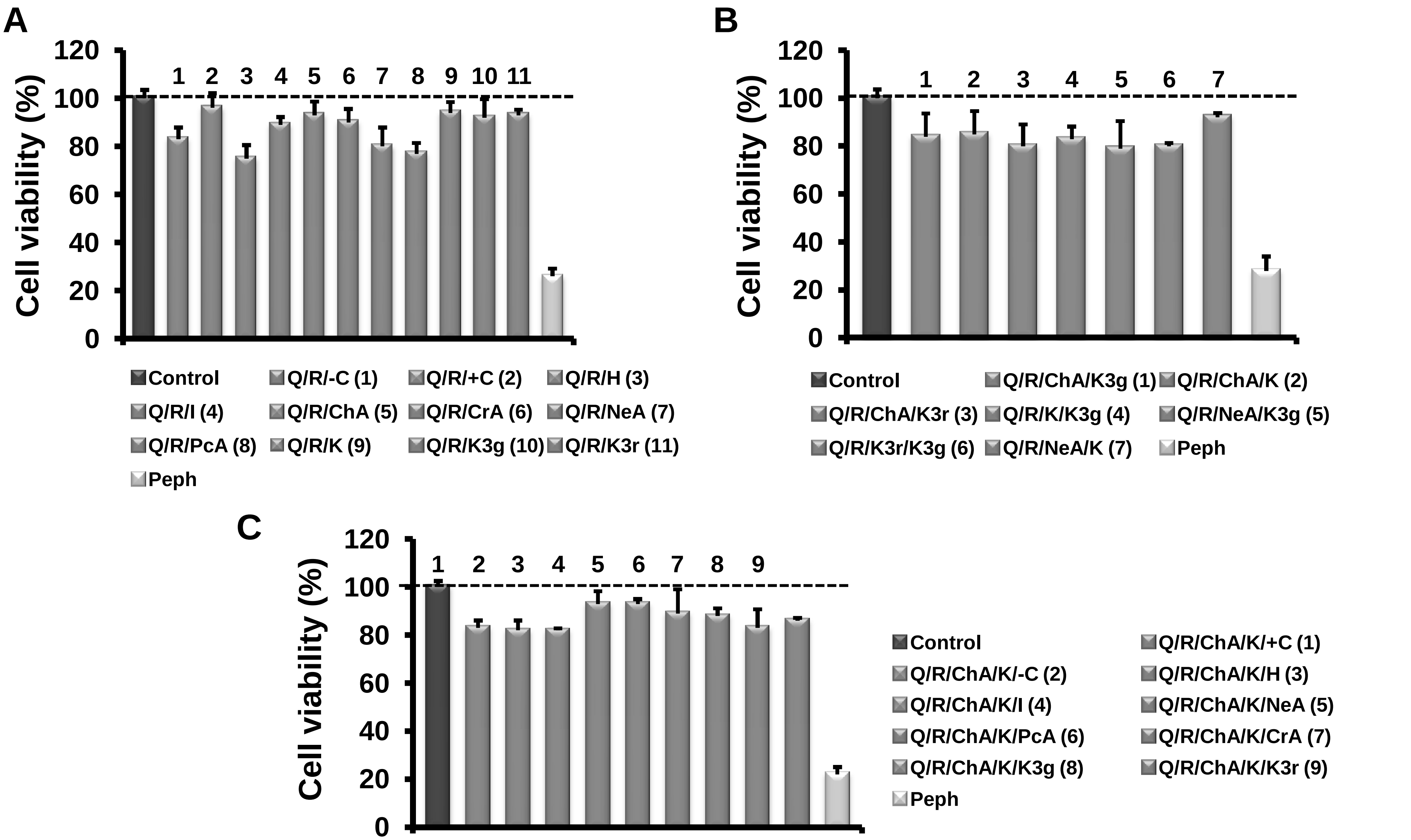

Fig S1. The effect of combination of Peph phenolic compounds on proliferation of LS174 cells compared to total Peph extract. Human colon adenocarcinoma LS174 cells were treated for 72h with different combinations (**A**. 3 combinations, **B**. 4 combinations, **C**. 5 combinations) of Peph phenolic compounds. All compounds where tested at equivalent concentrations to that present in 5µg/ml of the total peel polyphenolic extract. Cell viability was determined by MTT assay.
